# Homœopathic Medical College of Missouri

**Published:** 1893-04

**Authors:** 


### HOMŒOPATHIC MEDICAL COLLEGE OF MISSOURI.

The Homœopathic Medical College of Missouri conducted its 34th Annual Commencement, March 23d, at Pickwick Theatre. The invocation was asked by Rev. Charles B. Masden, of the Union M. E. Church, after which Dr. Wm. C. Richardson, Dean of the college, presented his report. He referred to the past year as one of the most prosperous in the history of the college. The institution, he said, is planning to increase its usefulness by the erection of a hospital, to be managed in connection with the college, during the coming summer. The degree of Doctor of Medicine was conferred by Dr. W. A. Edmonds, President of the Board of Trustees. In addition to instructions on professional ethics, Dr. Edmonds asked them to promise him to lead Christian lives and admonished them to abstain from the use of intoxicating liquors and tobacco.

